# Pterocarpan-Enriched Soy Leaf Extract Ameliorates Insulin Sensitivity and Pancreatic β-Cell Proliferation in Type 2 Diabetic Mice

**DOI:** 10.3390/molecules191118493

**Published:** 2014-11-13

**Authors:** Un-Hee Kim, Jeong-Hyun Yoon, Hua Li, Ji-Hyun Kang, Hyeon-Seon Ji, Ki Hun Park, Dong-Ha Shin, Ho-Yong Park, Tae-Sook Jeong

**Affiliations:** 1 Industrial Bio-materials Research Center, Korea Research Institute of Bioscience and Biotechnology (KRIBB), Daejeon 305-806, Korea; E-Mails: kuhalice@kribb.re.kr (U.-H.K.); hyun11-10@hanmail.net (J.-H.Y.); Leehua@kribb.re.kr (H.L.); bsloauclk@naver.com (J.-H.K.); gustjs@kribb.re.kr (H.-S.J.); hypark@kribb.re.kr (H.-Y.P.); 2 Department of Biomolecular Science, Korea University of Science and Technology, KRIBB, Daejeon 305-806, Korea; 3 College of Pharmacy, Chungnam National University, Daejeon 305-764, Korea; 4 Division of Applied Life Science, Gyeongsang National University, Jinju 660-701, Korea; E-Mail: khpark@gnu.ac.kr; 5 Insect Biotech Co. Ltd., Daejeon 305-811, Korea; E-Mail: dhshin@insectbiotech.co.kr

**Keywords:** *Glycine max*, insulin sensitivity, pancreas, pterocarpans, type 2 diabetes

## Abstract

In Korea, soy (*Glycine max* (L.) Merr.) leaves are eaten as a seasonal vegetable or pickled in soy sauce. Ethyl acetate extracts of soy leaves (EASL) are enriched in pterocarpans and have potent α-glucosidase inhibitory activity. This study investigated the molecular mechanisms underlying the anti-diabetic effect of EASL in C57BL/6J mice with high-fat diet (HFD)-induced type 2 diabetes. Mice were randomly divided into normal diet (ND), HFD (60 kcal% fat diet), EASL (HFD with 0.56% (wt/wt) EASL), and Pinitol (HFD with 0.15% (wt/wt) pinitol) groups. Weight gain and abdominal fat accumulation were significantly suppressed by EASL. Levels of plasma glucose, HbA1c, and insulin in the EASL group were significantly lower than those of the HFD group, and the pancreatic islet of the EASL group had greater size than those of the HFD group. EASL group up-regulated neurogenin 3 (*Ngn3*), paired box 4 (*Pax4*), and v-maf musculoaponeurotic fibrosarcoma oncogene homolog A (*MafA*), which are markers of pancreatic cell development, as well as insulin receptor substrate 1 (*IRS1*), *IRS2*, and glucose transporter 4 (*GLUT4*), which are related to insulin sensitivity. Furthermore, EASL suppressed genes involved in hepatic gluconeogenesis and steatosis. These results suggest that EASL improves plasma glucose and insulin levels in mice with HDF-induced type 2 diabetes by regulating β-cell proliferation and insulin sensitivity.

## 1. Introduction

Type 2 diabetes (T2D) is one of the fastest growing and most widespread diseases. The incidence of T2D has increased in recent years, and this trend is expected to continue because changing lifestyles have led to overconsumption of energy-rich foods, physical inactivity, and increased psychosocial stress. Shaw *et al.* predicted that the total number of patients with diabetes is expected to increase to 54% during the period from 2010–2030, which would require an annual growth rate of 2.2%, which is nearly twice the annual growth rate of the world adult population [1].

T2D is a metabolic disorder characterized by hyperglycemia resulting from defects in insulin secretion, action, or both [2]. Insulin regulates glucose homeostasis and is produced and secreted by pancreatic β-cells. Promotion of the proliferation and differentiation of pancreatic β-cells is an important component of T2D treatment [3,4]. Blood glucose levels are regulated by hepatic glucose production, which is controlled by insulin, and glucose uptake in peripheral tissue, which is stimulated by insulin. Under normal conditions, pancreatic β-cells secrete insulin at proper levels to maintain a normal blood glucose concentration. T2D is a result of insulin resistance associated with obesity and pancreatic β-cell dysfunction. In peripheral tissue, glucose enters cells through glucose transporter 4 (*GLUT4*) in a manner that is primarily controlled by insulin signaling [5,6]. Insulin signaling is mediated by the binding of insulin to its receptor, which leads to tyrosine phosphorylation of insulin receptor substrate 1 (*IRS1*) and *IRS2*. IRS1 phosphorylation enhances glucose transport and glycogen synthase activity [7,8]. Therefore, because blood glucose is controlled by insulin signaling, T2D therapy research is focused on insulin.

Soybean (*Glycine max*, also known as soy or soya bean) is cultivated globally, and its beneficial health effects include anti-cancer action [9,10], reduction in serum cholesterol [11,12], prevention of coronary heart disease [13,14], and immunomodulation [15,16]. While the bean of the soybean plant has been studied extensively, there is limited data on soy leaves. Soy leaves are consumed as a seasonal vegetable in the southern part of Korea, and their flavonoid and polyphenol profiles are different from those of soybeans. Soybeans contain major isoflavones, such as genistein, daidzein, glycitein, and their respective glycosides [17]. In contrast, soy leaves contain kaempferol glycosides and pterocarpans that are absent or present at low levels in soybeans [18,19].

The main components of ethyl acetate extracts of soy leaves (EASL) are pterocarpans, which are present in EASL at concentrations higher than those typically found in 95% ethanol extracts of soy leaves [19]. The pterocarpans coumestrol, glyceofuran, and phaseol strongly inhibit low-density lipoprotein (LDL) oxidation [20] and yeast α-glucosidase [19,21]. Pterocarpans produce cancer preventive [22,23], neuraminidase inhibitory [21], and anti-inflammatory [24] effects, but the effects of pterocarpans-enriched EASL against diabetes and obesity in animal models have not been reported.

Therefore, the main objective of this study was to investigate the effects of EASL in C57BL/6J mice with high-fat diet (HFD)-induced T2D, and to study the mechanisms underlying these effects, with a focus on gene expression involved in insulin sensitivity and β-cell proliferation, as well as cytokines. Pinitol (3-*O*-methyl-*chiro*-inositol) is a common constituent of legume plants and is a major component of the aqueous fraction of ethanolic extracts from soy leaves. Pinitol produces an insulin-like effect and improves insulin sensitivity [25], and so it was used as a positive control treatment in this study.

## 2. Results and Discussion

### 2.1. EASL Lowered Body Weight Gain and Abdominal White Adipose Tissue (WAT) Weight

The initial body weights of the four groups of mice were not significantly different. After a 12-week feeding period, the body weight of the HFD group (40.2 ± 0.8 g) was significantly greater than that of the normal diet (ND) group (26.8 ± 0.6 g). Meanwhile, body weights of the EASL group (36.3 ± 0.7 g) and the Pinitol group (36.7 ± 0.4 g) were significantly less than that of the HFD group. Compared with the HFD group, the supplementation with EASL or Pinitol did not affect food intake (Table 1).

**Table 1:** Effects of EASL supplementation on body weight gain and organ weights.

Parameter	ND	HFD	EASL	Pinitol
Body weight (g)				
Initial	20.3 ± 0.6 ^a^	19.5 ± 0.2 ^a^	20.1 ± 0.3 ^a^	19.6 ± 0.3 ^a^
Final	26.8 ± 0.6 ^c^	40.2 ± 0.8 ^a^	36.3 ± 0.7 ^b^	36.7 ± 0.4 ^b^
Weight gain (g/12 week)	6.5 ± 0.2 ^c^	20.7 ± 0.8 ^a^	16.3 ± 0.6 ^b^	17.0 ± 0.2 ^b^
Food intake (g/day)	2.5 ± 0.1 ^a^	2.1 ± 0.1 ^b^	2.1 ± 0.1 ^ab^	2.2 ± 0.1 ^ab^
Organ weight (g)				
Abdominal adipose tissue	0.26 ± 0.02 ^c^	2.01 ± 0.07 ^a^	1.50 ± 0.13 ^b^	1.80 ± 0.10 ^ab^
Pancreas	0.14 ± 0.02 ^a^	0.15 ± 0.03 ^a^	0.19 ± 0.01 ^a^	0.17 ± 0.01 ^a^
Liver	0.81 ± 0.01 ^b^	1.03 ± 0.04 ^a^	0.95 ± 0.02 ^a^	0.97 ± 0.01 ^a^
Muscle	0.31 ± 0.01 ^a^	0.31 ± 0.01 ^a^	0.31 ± 0.04 ^a^	0.32 ± 0.01 ^a^

Values are presented as mean ± SE (*n* = 10). ^a–c^ Means in the same row not sharing a common superscript letter are significantly different (*p* < 0.05) between the groups.

All mice were necropsied at the end of the experiment and organ weights were measured. WAT weight in the EASL group (1.50 ± 0.13 g) was significantly less than that of the HFD group (2.01 ± 0.07 g). However, there were no significant differences in the weights of the pancreas, liver, and muscle among the groups.

**Figure 1:**
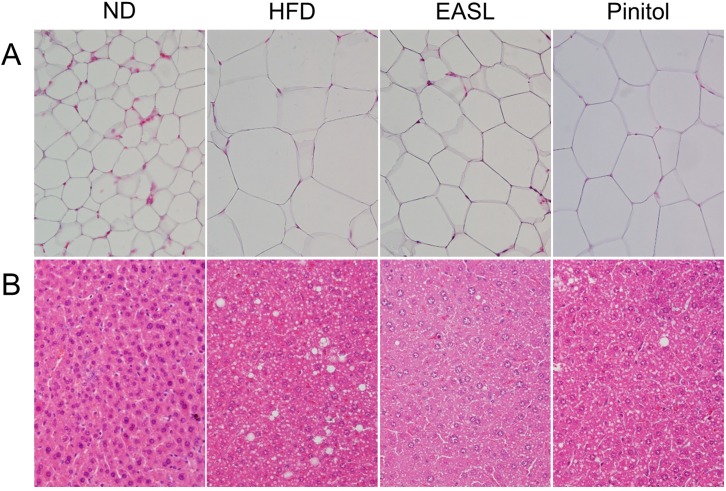
Histology of the white adipose tissue (WAT) (**A**) and liver (**B**) with hematoxylin and eosin (H&E) staining in HFD-fed C57BL/6J mice (400× original magnification).

The histological appearances of the WAT and the liver are shown in Figure 1. The adipocytes in the EASL-treated mice (Figure 1A) were markedly smaller than those of the HFD-fed mice. Although there were no differences in liver weight among the groups, the EASL group accumulated fewer hepatic lipid droplets than did the HFD group (Figure 1B).

### 2.2. Effect of EASL on Plasma Biochemical Parameters

At the end of the experiment, plasma levels of glucose, glycated hemoglobin (HbA1c), insulin, and triglyceride (TG) in HFD-fed mice were significantly higher than those of the ND mice (Table 2). Fasting glucose levels in EASL-treated mice were slightly lower than those of the ND mice, but the difference was not significant. The EASL treatment decreased the level of homeostasis model assessment of insulin resistance (HOMA-IR) index in HFD-fed mice, but the difference was not significant. The plasma HbA1c level of in the HFD group was 17.4% higher than that of the ND group, whereas treatment with EASL or Pinitol lowered the HbA1c level significantly to that of the ND group (*p* < 0.05). Furthermore, the plasma insulin level of the HFD group was threefold higher than that of the ND group, but the insulin level of the EASL-treated group was 40% less than that of the HFD group (*p* < 0.05). Consistent with this effect on HbA1c and insulin, after EASL supplementation, plasma total cholesterol (TC) and TG levels were 19.1% (*p* < 0.05) and 19.7% lower, respectively, than those of the HFD group. Additionally, plasma concentrations of glutamate oxaloacetate transaminase (GOT) and glutamate pyruvate transaminase (GPT) in EASL-supplemented mice tended to be significantly lower than those of the HFD mice. Pinitol showed a tendency to decrease fasting glucose (16.0%, *p* < 0.05), TC (41.7%, *p* < 0.05), and TG (26.0%) levels.

**Table 2:** Effects of EASL supplementation on plasma profiles.

Parameter	ND	HFD	EASL	Pinitol
Glucose (mg/dL)	93.9 ± 3.1 ^c^	203.7 ± 3.9 ^a^	193.0 ± 6.0 ^ab^	171.2 ± 9.1 ^b^
HbAlc (%)	4.6 ± 0.1 ^b^	5.4 ± 0.2 ^a^	4.5 ± 0.1 ^b^	4.5 ± 0.1 ^b^
Insulin (ng/mL)	0.5 ± 0.0 ^b^	1.5 ± 0.2 ^a^	0.9 ± 0.1 ^b^	0.9 ± 0.1 ^b^
HOMA-IR index	3.0 ± 0.1 ^b^	13.8 ± 2.5 ^a^	11.3 ± 1.1 ^a^	9.1 ± 1.9 ^ab^
TC (mg/dL)	149.7 ± 5.2 ^ab^	168.4 ± 5.6 ^a^	136.3 ± 11.0 ^b^	98.2 ± 9.6 ^c^
HDL-C/TC (%)	0.44 ± 0.05 ^a^	0.36 ± 0.08 ^a^	0.56 ± 0.10 ^a^	0.58 ± 0.04 ^a^
TG (mg/dL)	20.8 ± 3.9 ^b^	67.6 ± 11.4 ^a^	54.3 ± 9.1 ^a^	50.0 ± 5.4 ^a^
GOT (IU/L)	113.8 ± 10.0 ^a^	120.0 ± 8.1 ^a^	72.1 ± 1.2 ^b^	67.0 ± 1.9 ^b^
GPT (IU/L)	68.9 ± 4.7 ^ab^	71.4 ± 6.3 ^a^	54.8 ± 1.9 ^bc^	50.5 ± 2.3 ^c^

Values are presented as mean ± SE (*n* = 10). ^a–c^ Means in the same row not sharing a common superscript letter are significantly different (*p* < 0.05) between groups.

### 2.3. Effect of EASL on Pancreatic Function

**Figure 2:**
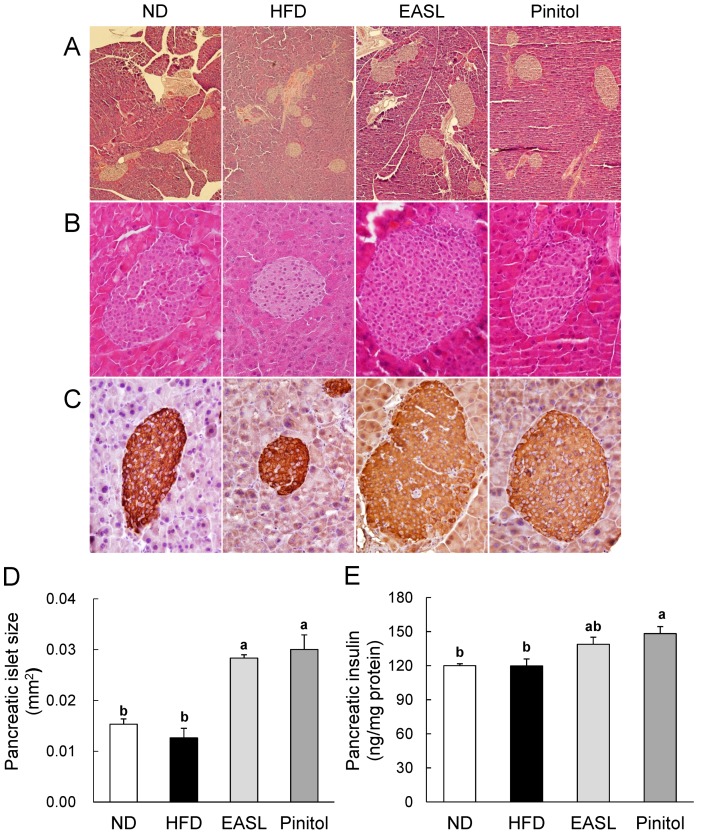
Effect of EASL supplementation on the pancreatic dysfunction in HFD-fed C57BL/6J mice. Histology of the pancreas with H&E (**A**,**B**) and insulin immunohistochemical (IHC) (**C**) staining. Magnification 100× (A) and 400× (B,C). The mean size of pancreatic islet (**D**) expressed in mm^2^. Pancreatic insulin levels (**E**). Pancreas was isolated and homogenized to measure pancreatic insulin levels. Values are presented as mean ± SE. ^a,b^ Means not sharing a common letter are significantly different between groups (*p* < 0.05).

The ameliorative effect of EASL on plasma HbA1c, insulin, and glucose levels may have been due to its prevention of β-cell dysfunction in pancreatic islets. Therefore, we investigated the histology of the pancreas via H&E and insulin immunohistochemical (IHC) staining. In the pancreas of the ND and HFD groups had intermediate-sized islets, whereas the pancreas of EASL and Pinitol groups had large-sized islets (Figure 2A,B). On the other hand, the insulin were sparsely expressed in the pancreatic islet of the EASL group compared with the ND and HFD group as demonstrated by insulin IHC staining (Figure 2C). For interpretation of the abnormal observation, we examined the mean size of the pancreatic islet and the pancreatic insulin content. While the pancreatic insulin content of the HFD group was similar to that of the ND group, the islet size of the HFD group was smaller than that of the ND group. We inferred that these results were associated with the increased number of the pancreatic islet in HFD group corresponding to Figure 2A. (Figure 2D,E). Kou *et al.* suggest that islet number rather than islet size is a major determinant of β-cell mass in humans [26]. In this study, we measured pancreatic insulin content instead of β-cell mass or β-cell number. The insulin content and islet size of the pancreas were not increased by the HFD as part of compensatory adaptation in an insulin-resistant state to maintain normoglycemia. (Figure 2 D,E). Peyot *et al.* reported that HFD (60 kcal% fat)-fed C57BL/6 mice show β-cell maladaptation caused by insulin resistance with resulting dysfunction and mild hyperglycemia [27]. In the present study, HFD-fed mice for 12 weeks did not reveal the pancreatic β-cell compensation owing to hyperglycemia and hyperinsulinemia according to the plasma profile. Nevertheless, the pancreatic islet size and insulin content in EASL-supplemented mice were increased for compensation to maintain normoglycemia compared with those of the HFD mice. The plasma levels of insulin, HbA1c, and glucose in the EASL and Pinitol groups were lower than that of the HFD group. These results suggest that the EASL dietary supplementation may enhance pancreas function and insulin sensitivity.

**Figure 3:**
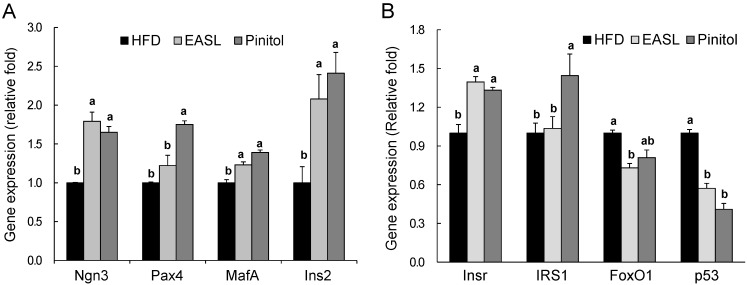
Effects of EASL supplementation on the relative mRNA expression levels of pancreatic genes related to β-cell development (**A**) or insulin sensitivity (**B**). The levels of mRNA in pancreatic tissue were measured by real-time quantitative RT-PCR (qRT-PCR) and normalized to glyceraldehyde-3-phosphate dehydrogenase (*GAPDH*) expression. Values are presented as mean ± SE. ^a,b^ Means not sharing a common letter are significantly different between groups (*p* < 0.05).

Next, we investigated whether EASL influenced the expression of pancreatic β-cell proliferation-related genes, such as neurogenin 3 (*Ngn3*), paired box 4 (*Pax4*), v-maf musculoaponeurotic fibrosarcoma oncogene homolog A (*MafA*), and insulin II (*Ins2*). The mRNA levels of *Ngn3*, *MafA*, and *Ins2* in the EASL group were significantly higher than those of the HFD group (Figure 3A). We also examined levels of key molecules in the insulin sensitivity. Compared with the HFD group, supplementation with EASL significantly increased insulin receptor (*Insr*) mRNA expression (Figure 3B) and significantly reduced the transcriptional expression of the β-cell apoptosis-related genes forkhead box O1 (*FoxO1*) and *p53* in pancreatic tissue. Pinitol group showed mRNA expression of *Ngn3*, *Pax4*, *MafA*, *Ins2*, *Insr*, and insulin receptor substrate 1 (*IRS1*) that was significantly greater than that of the HFD group (Figure 3A,B).

### 2.4. Effect of EASL on Expression of Gluconeogenesis and Insulin Sensitivity-Related Genes in Liver Tissue and WAT

**Figure 4:**
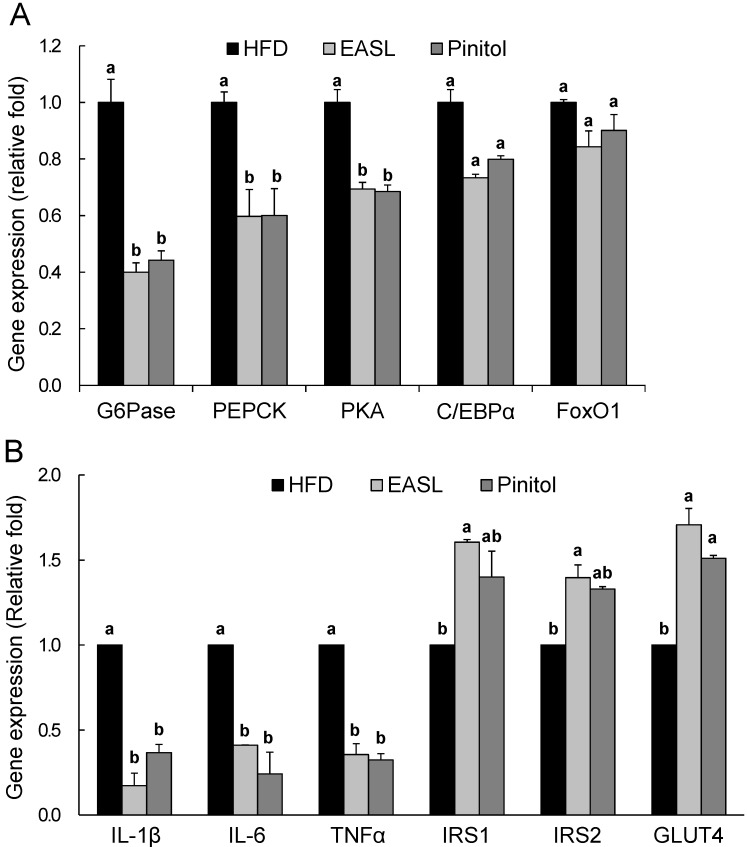
Effects of EASL dietary supplementation on the relative mRNA expression levels of genes in the liver and WAT. Expression was measured by qRT-PCR for genes involved in gluconeogenesis (**A**) and insulin sensitivity (**B**) and then normalized to *GAPDH* expression. Values are presented as mean ± SE. ^a,b^ Means not sharing a common letter are significantly different between groups (*p* < 0.05).

The mRNA levels of hepatic genes involved in gluconeogenesis were measured by real-time qRT-PCR analysis. HFD-induced elevations in expression of glucose-6-phosphatase (*G6Pase*) and phosphoenolpyruvate carboxykinase (*PEPCK*) genes were markedly suppressed by dietary EASL or pinitol supplementation. The expression level of upstream transcription factor protein kinase A (*PKA*) in the EASL group was significantly lower than those of the HFD group. *FoxO1* and CCAAT/enhancer binding protein alpha (*C/EBPα*) mRNA abundance were reduced, but the difference was not significant (Figure 4A).

Next, we evaluated EASL-induced changes in the expression of WAT genes associated with insulin signaling pathways and cytokines via qRT-PCR analyses. The expression levels of cytokines interluekin-1 beta (*IL-1β*), interleukin-6 (*IL-6*), and tumor necrosis factor alpha (*TNFα*) in the WAT from the EASL-treated group (82.8%, 58.9%, and 64.4%, respectively) were significantly lower than those measured in the WAT of the HFD group. Furthermore, mRNA levels of *IRS1*, *IRS2*, and glucose transporter 4 (*GLUT4*) in the EASL-treated group were significant higher than those of the HFD group (Figure 4B). Changes in gene expression similar to those mentioned above for EASL also occurred in the pinitol-supplemented positive control group.

### 2.5. Discussion

Soybean is a globally exploited food crop that is relatively high in protein, and the health effects of the soy bean have been exhaustively studied. However, the leaves of the soybean plant are consumed in parts of Asia, and the health effects of soy leaves are not as well-known as those of soy beans. In recent clinical studies, soy leaf extracts produced body fat loss, and lowered fasting blood glucose, plasma TC, and HbA1c levels [28,29]. Therefore, we hypothesized that dietary supplementation with pterocarpan-enriched soy leaf extracts might produce therapeutic effects against T2D associated with obesity. Insulin resistance because of obesity is the most important etiological factor in the onset of T2D, in parallel with its central role in the pathogenesis of metabolic syndrome, which is a cluster of conditions that increase the risk of diabetes, heart disease, and stroke [8]. We supplemented a diabetes-inducing HFD with pterocarpan-enriched extract from soy leaves in C57BL/6J mice for 12 weeks to investigate whether the extract prevented T2D and obesity produced by impaired glucose tolerance.

As expected, the HFD group was induced obesity and T2D, which were accompanied by abnormally high weight, greater adipocyte size, and elevated levels of plasma lipids, glucose, HbA1c, and insulin. Plasma analysis revealed that EASL significantly improved the plasma profile; although the glucose level was not significantly changed by EASL supplementation, plasma HbA1c and insulin levels were markedly lower than the levels of the HFD group (Table 2). HbA1c and insulin levels are important parameters in the diagnosis of T2D and diabetic complications. The size of the pancreatic islets of the EASL group was greater than that of the islets of the HFD group (Figure 2). Accordingly, the prevention of β-cell dysfunction in the pancreatic islets of the EASL group was a result of lowered levels of plasma HbA1c, insulin, and glucose. EASL supplementation produced plasma TC levels markedly lower than those of the HFD group. Decreased plasma TC and TG concentrations were verified by the reduction in WAT weight and adipocyte size. In addition, EASL treatment inhibited weight gain and the formation of lipid droplets within hepatocytes. Moreover, plasma GOT and GPT levels in the EASL group, which are indicative of the level of liver function, were dramatically lower than those of the HFD group.

The beneficial effects of lowered HbA1c and glucose levels may be mediated by the prevention of β-cell dysfunction in pancreatic islet. Therefore, we evaluated the expression of genes involved in β-cell proliferation and differentiation. *Ngn3* is a transcription factor that is essential for pancreatic endocrine cell differentiation and development. In the pancreas, α-, β-, and δ-cells are differentiated from *Ngn3*-expressing precursors. The transcription factor *Pax4* also plays an important role in the differentiation of β- and δ-cells [30]. *MafA*, a strong activator of insulin gene transcription, is expressed during the final stage of β-cell differentiation [31]. Recent findings have suggested that it is possible to utilize small molecules or other techniques to reprogram cells into a β-cell-like state by expressing transcription factors, such as *Ngn3*, *Pax4*, pancreatic and duodenal homeobox 1 (*PDX1*), and *MafA* [32,33]. We found that mRNA levels of *Ngn3* and *MafA* were significantly increased in the EASL group (Figure 3A). In addition, in pancreatic β-cells, insulin signaling pathways are reported to play an important role in the maintenance of cellular function [34]; insulin signaling in β-cells is important for insulin secretion, proliferation, and β-cell survival. EASL dietary supplementation significantly increased *Insr* transcript expression, but expression level of *IRS1* was not changed. In addition, expression of the β-cell apoptosis-related gene *p53* and *FoxO1* were markedly reduced by dietary EASL supplementation (Figure 3B). These results suggest that EASL ameliorates insulin sensitivity by enhancing pancreatic β-cell proliferation and function.

The insulin-like effect of pinitol was mediated by reduced glucose and HbA1c levels and increased glucose uptake [35]. Dietary supplementation with pinitol ameliorates insulin sensitivity by positively regulating insulin signaling [35,36]. In addition to this insulin-like effect, pinitol produces anti-hyperlipidemic, cardioprotective, and anti-oxidative effects [37,38,39]. In this study, we confirmed the anti-diabetic effect of pinitol, which produced an effect similar to that of EASL (Figure 3A,B).

Reduced insulin levels may be caused by improved insulin sensitivity in insulin target organs, such as the liver, skeletal muscle, and adipose tissue. The liver is extremely sensitive to insulin, which controls its plasma glucose regulating function. Usually, Insr activation induces *IRS1* Tyr phosphorylation, thereby triggering phosphoinositide 3-kinase (*PI3K*)-protein kinase B (*PKB*) activation. *PKB* controls the expression of gluconeogenic and lipogenic enzymes by regulating the activity of the winged helix, or forkhead (*Fox*), class of transcription factors [40]. It is well known that *PKA-cAMP* response element binding protein (*CREB*) and *FoxO1* signaling mediates the expression of genes involved in gluconeogenesis. In a fasted state, *CREB* phosphorylation is induced by *PKA* and *C/EBPα*, and it acts as a major transcription factor in the control of hepatic gluconeogenesis [41]. *PKA* phosphorylates *CREB*, and the action of *CREB* appears to be limited primarily by nuclear entry of the *PKA* catalytic subunit. Activated *CREB* directly stimulates the expression of gluconeogenic genes, such as *G6Pase* and *PEPCK* [42]. In addition, gluconeogenesis is suppressed by insulin signaling that leads to phosphorylation of *FoxO1* when the organism is in a fed state [43]. In this study, it is interesting that EASL significantly decreased mRNA levels of *G6Pase* and *PEPCK*, implying decreased hepatic gluconeogenesis (Figure 4A). To further evaluate these effects of EASL on gene expression and gluconeogenesis, we examined *PKA*, and *C/EBPα, and FoxO1* gene expressions, which is upstream of hepatic gluconeogenic genes. *PKA* mRNA expression was dramatically down-regulated by dietary supplementation with EASL (*p* < 0.05). In addition, EASL treatment suppressed *C/EBPα* and *FoxO1* transcript levels, but these changes were not significant (Figure 4A). These results suggest that dietary supplementation with EASL might reduce gluconeogenesis by enhancing insulin sensitivity, and regulate the hepatic glucose homeostasis, which leads to lowering of glucose level.

Adipose tissue, the typical insulin-target organ, can respond quickly to excess caloric intake through adipocyte hypertrophy and hyperplasia, and enlarged adipose tissue results in altered production of various adipokines and inflammatory chemokines and cytokines, which impacts insulin sensitivity [44]. In obesity, increased accumulation of adipose tissue is accompanied by chronic adipose inflammation, which seems to play an important role in the pathogenesis of obesity-related insulin resistance [45]. It has been reported that increased cytokine levels inhibit the normal action of insulin. In particular, the well-known pro-inflammatory cytokines, *IL-1β*, *IL-6*, and *TNFα* are secreted at high levels in the visceral adipose tissue [44]. Plasma *IL-1β*, *IL-6*, and *TNFα* levels are typically elevated in the metabolic syndrome and are associated with obesity, insulin resistance, and T2D. Specifically, *IL-6* production in adipose tissue is increased by obesity, related to insulin resistance, and predictive of the incidence of T2D [46,47]. *TNFα* induces production of chemokines and adhesion molecules in the adipose tissue that serve as markers of inflammation; therefore, *TNFα* mediates adipose tissue inflammation [48]. In this study, EASL inhibited dramatically transcriptional expression of *IL-1β*, *IL-6* and *TNFα* in WAT. In addition, EASL enhanced expression of insulin sensitivity-related genes *IRS1*, *IRS2*, and *GLUT4* (Figure 4B). These results suggest that insulin sensitivity may be enhanced by decreased cytokine production in the WAT.

## 3. Experimental Section

### 3.1. Preparation of Soy Leaf Extracts

Soybeans, *Glycine max* (L.) Merr., were cultivated in Jeungpyeong County, Chungcheongbuk-do, Korea for 4 months, after which soy leaves were collected and immediately air-dried at room temperature for 5 days. The dried soy leaves were chopped with the aid of a laboratory blade cutter and stored at 4 °C. The chopped soy leaves (500 g) were extracted with 5 L ethyl acetate (EtOAc) at room temperature for 3 days. The extract was filtered and evaporated to dryness under reduced pressure at 40 °C to obtain the EASL (33.1 g).

### 3.2. HPLC Analysis of EASL

**Figure 5:**
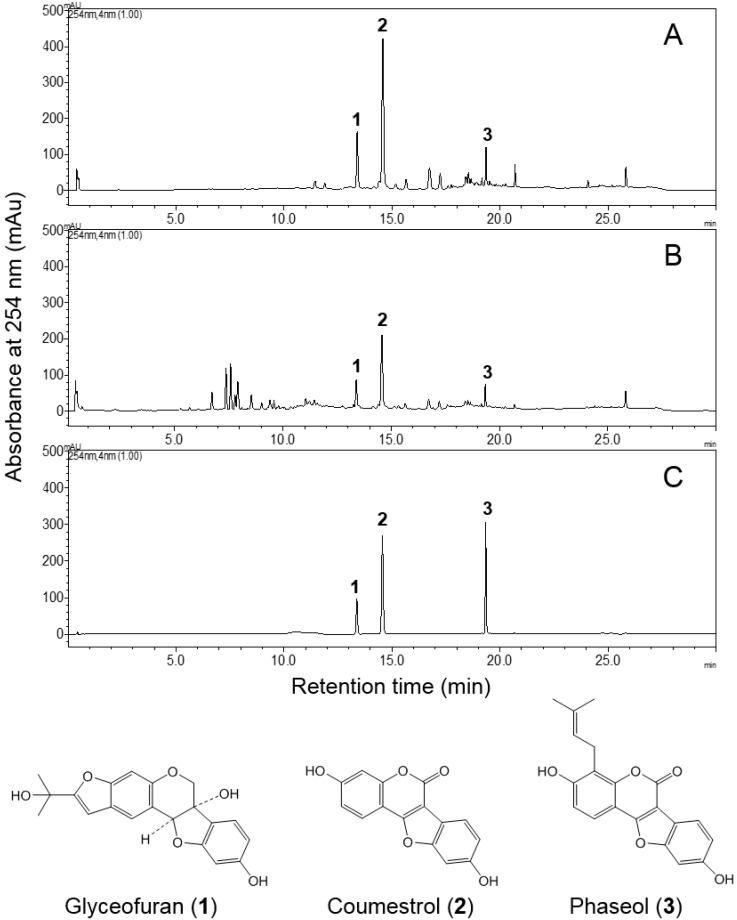
HPLC chromatograms of EASL (**A**), 95% EtOH extract (**B**) from soy leaves, and the external standards **1**, **2**, and **3** (**C**) and their chemical structures. The HPLC chromatograms were detected at 254 nm. Peaks: **1**, glyceofuran (T_R_: 13.36 min); **2**, coumestrol (T_R_: 14.55 min); and **3**, Phaseol (T_R_: 19.33 min). The mean peak areas of **1**, **2**, and **3** were 12.5%, 36.6%, and 5.4%, respectively, in EASL; the mean peak areas of **1**, **2**, and **3** were 6.2%, 19.5%, and 3.3%, respectively, in 95% ethanol extract.

The main polyphenol compounds present in EASL (20 mg/mL) and 95% EtOH extract (20 mg/mL) of soy leaves were analyzed and confirmed using a Shimadzu HPLC system equipped with a binary pump delivery system, a photo diode array detector, and an autosampler (Shimadzu Corp., Tokyo, Japan) and a Brownlee SPP C18 column (4.6 × 50 mm, 2.7 µm; PerkinElmer, Inc., Waltham, MA, USA). The injection volume was 5 µL and the mobile phase was 0.1% acetic acid in water (solvent A) and acetonitrile (solvent B). The linear gradient elution program was as follows: 5%–30% B for 0–15 min, 30%–80% B for 15–20 min, 80%–100% B for 20–23 min, 100% B for 23–27.5 min, and 100%–5% B for 27.5–30 min. The flow rate was 1.8 mL/min and the absorbance was 254 nm. Coumestrol obtained from Sigma-Aldrich (St. Louis, MO, USA) and glyceofuran, and Phaseol, isolated from EASL by Ki Hun Park (Gyeongsang National University) [20], were used as external standards of HPLC analysis. The peak areas of the major pterocarpan components glyceofuran (**1**), coumestrol (**2**), and phaseol (**3**) in the EASL (56.4% ± 0.3%) were about 1.9-fold greater than those of the 95% ethanol extract (29.0% ± 0.3%), which was prepared for preliminary study (Figure 5).

### 3.3. Animals and Diets

Male C57BL/6J (4-week-old) mice were purchased from Japan SLC (Shizuoka, Japan) and housed in a facility with controlled temperature (25 ± 2 °C), humidity (50% ± 5%), and lighting (alternating 12-h periods of light and dark) at the Korea Research Institute of Bioscience and Biotechnology (KRIBB), in Daejeon, Korea. Animals were fed a standard normal laboratory diet (Taklad 2018S, Harlan Laboratories, Inc., Indianapolis, IN, USA) for an acclimation period of 2 weeks, after which the mice were divided into 4 groups of 10 mice each: the ND group, which was fed a standard normal diet; the HFD group, which was fed a 60 kcal% fat diet (D12492, Research Diets, Inc., New Brunswick, NJ, USA) with no supplement; the EASL group, which was fed a HFD with 0.56% EASL (wt/wt); or Pinitol group, which was fed a HFD with 0.15% pinitol (wt/wt) (CnF Co. Ltd., Seoul, Korea) as a positive control group. The groups of mice were allowed to consume their respective diets for a 12-week experimental period. Animal study protocols were approved by the Animal Care Committee of KRIBB (KRIBB-ACE-13028).

### 3.4. Measurement of Metabolic Parameters

Body weight and food intake were monitored every week during the experiment. At the end of the experimental period, all mice were anesthetized with ether after a 12-h fast. Blood was collected from the inferior vena cava into an EDTA-coated tube. The collected blood samples were centrifuged at 800 g at 4 °C for 15 min and stored at −72 °C. The plasma concentrations of glucose, TC, TG, and high-density lipoprotein-cholesterol (HDL-C) were analyzed using an automatic blood chemical analyzer (Hitachi-720, Hitachi, Ltd., Tokyo, Japan). HbA1c and insulin levels were determined using an HbA1c Measuring System (Infopia, Anyang, Korea) and an ELISA kit (Alpco Diagnostics, Windham, NH, USA), respectively. The plasma GOT and GPT levels were measured using commercially available kits (Asan Pharm. Co., Seoul, Korea). The HOMA-IR index was calculated as described: HOMA-IR index = [fasting insulin concentration (ng/mL)] × 24.8 × [fasting glucose concentration (mg/dL)]/405 [49]. The WAT, liver, and pancreas were removed, weighed, and frozen in liquid N_2_ or stored in RNAlater^TM^ (Qiagen, Valencia, CA, USA) for further analysis.

### 3.5. Histological Analysis of WAT, Liver, and Pancreas

Freshly isolated WAT, liver, and pancreas sections were fixed in 10% formalin for 24 h for histopathological analysis. Following a rinse in flowing water, tissues were processed in a paraffin automatic processor using a programmed cascade. The paraffin-embedded samples were sliced into 5-µm thick sections and stained with hematoxylin and eosin (H&E). The mean size of pancreatic islet were measured using MetaMorph Imaging System (Meta Imaging Software, Sunnyvale, CA, USA).

### 3.6. IHC Analysis of the Pancreas

Paraffin-embedded sections of the pancreas were deparaffinized in xylene, hydrated in graded ethanol solutions, and boiled in 10 mM sodium citrate buffer (pH 6.0) for 5 min for antigen retrieval. IHC staining for insulin was performed using the VECTASTAIN Elite ABC kit (Vector Laboratories, Burlingame, CA, USA). Sections were treated with blocking serum and incubated with anti-insulin antibody (1:200, Santa Cruz Biotechnology, Santa Cruz, CA, USA) at 4 °C overnight. Biotinylated goat anti-rabbit IgG was used as the secondary antibody. Antibody reactivity was developed with a diaminobenzidine tetrahydrochloride (DAB) peroxidase substrate kit (Vector Laboratories).

### 3.7. Pancreatic Insulin Content

To measure pancreatic insulin contents, pancreas was cut in half, weighed, and then added into 70% EtOH solution containing 1.5% HCl. Tissue was homogenized by TissuLyser (Qiagen, Valencia, CA, USA), and incubated in the same solution overnight at 4 °C. The tissue was then centrifuged at 300× g for 15 min at 4 °C and the supernatant neutralized with 1 M Tris buffer (pH 7.5). Insulin contents were determined by ELISA kit (Alpco Diagnostics, Windham, NH, USA).

### 3.8. Real-Time Quantitative RT-PCR (qRT-PCR)

After isolation of total RNA from pancreas, liver, and abdominal adipose tissue using the RNeasy mini kit (Qiagen, Valencia, CA, USA) as described by the manufacturer, cDNA was prepared from total RNA (1 µg) using the High-capacity cDNA reverse transcription kit (Applied Biosystems, Inc., Foster City, CA, USA). Real-time qRT-PCR was performed using SYBR Green Supermix reagent (Roche, Mannheim, Germany) with an Applied Biosystems^®^ 7500 Real Time PCR system (Life Technologies, Grand Island, NY, USA). The cycling conditions were 95 °C for 10 min, followed by 50 cycles of 95 °C for 10 s, 60 °C for 20 s, and 72 °C for 50 s. To detect and eliminate possible primer-dimer artifacts, a dissociation curve was generated by adding a cycle of 95 °C for 15 s, 60 °C for 1 min, and 95 °C for 15 s. Results were normalized using glyceraldehyde-3-phosphate dehydrogenase (*GAPDH*) as a reference gene. The primers used in the experiments are shown in Table 3.

**Table 3:** Sequences of the primers used in qRT-PCR.

Gene	Forward Primer	Reverse Primer
*C/EBPα*	CAAGAAGTCGGTGGACAAGA	TCAACTCCAGCACCTTCTGT
*FoxO1*	TGGGCCCTAATTCGGTCAT	TTGGGTCAGGCGGTTCATAC
*GLUT4*	GCCCCACAGAAGGTGATTGA	AGCGTAGTGAGGGTGCCTTGT
*G6Pase*	GCTGGAGTCTTGTCAGGCATT	AAACAAGAAGATGGTGATGAGACAAT
*GAPDH*	ACATCATCCCTGCATCCACT	AGATCCACGACGGACACATT
*Ins2*	CCCCACCCAGGCTTTTGT	GCGGGACATGGGTGTGTAG
*Insr*	CTGAACAAAGATGACAACGAGGAA	CTTACAGATGGTTGGGCAAACTT
*IRS1*	GAGAAGAGACTGGCTCGGAAGA	GCCTATTCTGCCCAACTCAACT
*IRS2*	GGCCCGAACCTCAATAACAA	CCGCGCAACACGAAAAAG
*IL-1β*	ATGAGGACATGAGCACCTTC	CATTGAGGTGGAGAGCTTTC
*IL-6*	GCTACCTGGAGTACATGAAG	CTGTGACTCCAGCTTATCTG
*MafA*	GAACCGGAGGAGAATAAGAGG	AGTCAAGTTGAGCATCACTGC
*Ngn3*	CACTCAGCAAACAGCGAAGAAG	GTCAGTGCCCAGATGTAGTTGTG
*p53*	ACATCATCCCTGCATCCACT	AGATCCACGACGGACACATT
*Pax4*	CCCAGTGTGTCCTCTATCAATCG	GGGAAGAACTGGAGCCAACA
*PEPCK*	AGACCCTGCGAGTGCTTAGTG	AGGGTCAGTGTGGCAGTATTCTC
*PKA*	GGTTTTGCCAAGCGTGTGA	CAGCCTTGTTGTAGCCTTTGC
*TNFα*	CTCAGATCATCTTCTCAAAATTCGAGTGACA	CTTCACAGAGCAATGACTCCAAAGT

### 3.9. Statistical Analysis

Data from tissues and plasma are presented as mean ± SE. Significant differences among the groups were determined by one-way analysis of variance (ANOVA) using JMP^®^ software (SAS Institute Inc., Cary, NC, USA). Statistical analysis was performed using Student’s *t*-test. Values of *p* < 0.05 were considered significant.

## 4. Conclusions

Our results show that pterocarpan-enriched EASL enhanced the action of insulin by improving the structure of pancreatic islets of Langerhans in insulin target organs, as well as the liver and WAT, and thereby reduced plasma glucose and insulin levels and body weight gain. In addition, EASL increased the size of pancreatic islets and mRNA expression of β-cell proliferation-related genes. EASL modulated the expression of genes involved in hepatic gluconeogenesis, decreased hepatic steatosis, and improved plasma lipid profiles in mice. These results suggest that EASL dietary supplementation can prevent insulin resistance and β-cells dysfunction induced by a high-fat diet.
